# Restructuring of an Ir(210) electrode surface by potential cycling

**DOI:** 10.3762/bjnano.5.148

**Published:** 2014-08-25

**Authors:** Khaled A Soliman, Dieter M Kolb, Ludwig A Kibler, Timo Jacob

**Affiliations:** 1 Institut für Elektrochemie, Universität Ulm, 89069 Ulm, Germany; 2 Permanent address: Electrochemistry and Corrosion Laboratory, Physical Chemistry Department, National Research Centre, Cairo, 12622, Egypt

**Keywords:** CO adlayer oxidation, cyclic voltammetry, Ir(210) single crystal, potential cycling, scanning tunnelling microscopy, surface restructuring

## Abstract

This study addresses the electrochemical surface faceting and restructuring of Ir(210) single crystal electrodes. Cyclic voltammetry measurements and in situ scanning tunnelling microscopy are used to probe structural changes and variations in the electrochemical behaviour after potential cycling of Ir(210) in 0.1 M H_2_SO_4_. Faceted structures are obtained electrochemically as a function of time by cycling at a scanrate of 1 V·s^−1^ between −0.28 and 0.70 V vs SCE, i.e., between the onset of hydrogen evolution and the surface oxidation regime. The electrochemical behaviour in sulfuric acid solution is compared with that of thermally faceted Ir(210), which shows a sharp characteristic voltammetric peak for (311) facets. Structures similar to thermally-induced faceted Ir(210) are obtained electrochemically, which typically correspond to polyoriented facets at nano-pyramids. These structures grow anisotropically in a preferred direction and reach a height of about 5 nm after 4 h of cycling. The structural changes are reflected in variations of the electrocatalytic activity towards carbon monoxide adlayer oxidation.

## Introduction

The surface structure of metal electrodes is a decisive factor for kinetics of many electrochemical processes and electrocatalytical reactions [1–3]. Since the behaviour of polycrystalline material is often quite complex, relations between the surface structure of an electrode and its activity for a given reaction are typically investigated in experiments by using clean and well-defined model systems, such as single crystal surfaces [4–6], epitaxially grown monolayers [7–8] or preferentially-shaped nanoparticles [9]. In all cases, detailed protocols have been established for the reproducible preparation of these model electrodes, which have been extensively characterized in recent years.

The development of so-called electrochemical surface science has shown that the geometric surface structure of metals is often identical under ultrahigh-vacuum (UHV) conditions and in contact with an electrolyte. However, there are several examples for which the stability of electrode surfaces is limited to certain potential regions or reaction conditions. Among these are (i) reconstructed surfaces of Au and Pt single crystals [10–11], (ii) structural changes in operando, e.g., for hydrogen evolution at PdAu nanoparticles [12], (iii) removal of islands by adsorbates, such as electrochemical annealing of Au(100) by adsorbed chloride [13–14], (iv) dissolution of metals at positive potentials and restructuring by oxidation–reduction cycles [15–16]. Thus, morphological changes between thermodynamically stable structures can be induced for example by temperature, electrode potential or specific adsorption.

Unlike reconstruction phenomena, the faceting of surfaces leads to structures, which exist in the bulk lattice already. In earlier studies, we have examined the electrochemical behaviour of Ir single crystals [17–18], including thermally-induced faceted Ir(210) [19–20]. Besides the laborious preparation under UHV conditions [21–22], faceted Ir(210) can easily be obtained outside a UHV chamber by inductive heating and cooling in nitrogen gas atmosphere [19–20]. Such thermally-induced faceted Ir(210) has been characterized by cyclic voltammetry and in situ scanning tunnelling microscopy (STM) [20]. Thus, very similar surface structures with nanometer-scale pyramids consisting of (110) and {311} facets could be prepared in- and outside a UHV chamber. It was found that the presence of oxygen is crucial for the faceting process on Ir(210) [21–22].

Theoretical calculations for the Ir(210) system, based on first principles, provided supportive information. It was shown that, due to the anisotropy in surface free energy for the different Ir surface orientations, the adsorption of more than 0.5 ML oxygen causes the formation of nano-pyramids exhibiting (110) and {311} faces to be thermodynamically more stable than the original Ir(210) substrate [19,23]. Based on density functional theory calculations it was predicted that the faceting process of Ir(210) can also be induced by the electrode potential [19].

Here, we present a combined electrochemical and in situ STM study of Ir(210), which demonstrates that the faceted surface is not only stable in a certain potential region, but can also be obtained electrochemically. The simple polarization of Ir(210) at positive potentials did not lead to the formation of facets. However, potential cycling into the surface oxidation potential region leads to a restructuring of the Ir(210) surface. Carbon monoxide adlayer oxidation was chosen as a structure-sensitive reaction to study the electrocatalytic activity of restructured Ir(210) surfaces compared to non-restructured Ir(210).

## Experimental

A cylindrical Ir(210) single crystal (4 mm in diameter and thickness, MaTecK Jülich, Germany) was used both for electrochemical and in situ STM investigations. Before each measurement, the single crystal was annealed at 1700 °C by inductive heating in a stream of nitrogen gas (5.0, MTI IndustrieGase AG, Neu-Ulm, Germany) mixed with carbon monoxide (4.7, MTI) or hydrogen (5.0, MTI). The annealing temperature was controlled (contact-free) by an infrared pyrometer (Infratherm IGAR 12-LO, IMPAC Infrared GmbH, Frankfurt am Main, Germany). After short cooling in the same gas mixture, the single crystal was transferred under nitrogen atmosphere to the electrochemical cell. The crystal was immersed under potential control into 0.1 M H_2_SO_4_ at −0.1 V vs SCE and brought to a stable hanging-meniscus configuration. The CO adlayer was anodically stripped in a single voltammetric scan up to 0.7 V. Surface quality and cleanliness were assured by recording reproducible current–potential curves in the hydrogen adsorption region. Subsequently, the crystal was transferred to the STM cell, while a droplet of electrolyte protected its surface. The solutions were prepared from H_2_SO_4_ (Merck, suprapur) and ultrapure water (18.2 MΩ·cm at 25 °C, total oxidizable carbon < 1 ppb as recorded with an A10 TOC Monitor, Millipore). The electrolytes were purged with nitrogen gas. The electrochemical measurements were performed in a conventional three-electrode glass cell. A saturated calomel electrode (SCE) and a Pt wire were used as the reference and counter electrodes, respectively. Pt wires were used for the STM cell as counter and pseudo-reference electrodes. The STM images were recorded with a Digital Instruments Nanoscope III (Digital Instruments, Santa Barbara, California). For the preparation of the STM tips, a Pt/Ir wire (80/20) was etched in 4.5 M NaCN and coated with an electrophoretic paint to reduce Faradic currents at the tip/electrolyte interfaces below 50 pA.

## Results and Discussion

### Electrochemical behaviour of Ir(210)

Annealing and cooling of Ir(210) in a nitrogen gas atmosphere containing trace amounts of oxygen was shown earlier to induce surface faceting, which can be avoided by adding a reducing gas, such as hydrogen [19–20]. In this study, CO was mixed to the cooling gas in order to start with a non-faceted surface. In this case, a CO adlayer is formed on the Ir surface, which survives the transfer to the electrochemical cell and which can easily be stripped of at positive potentials, as described in section Experimental.

**Figure 1:**
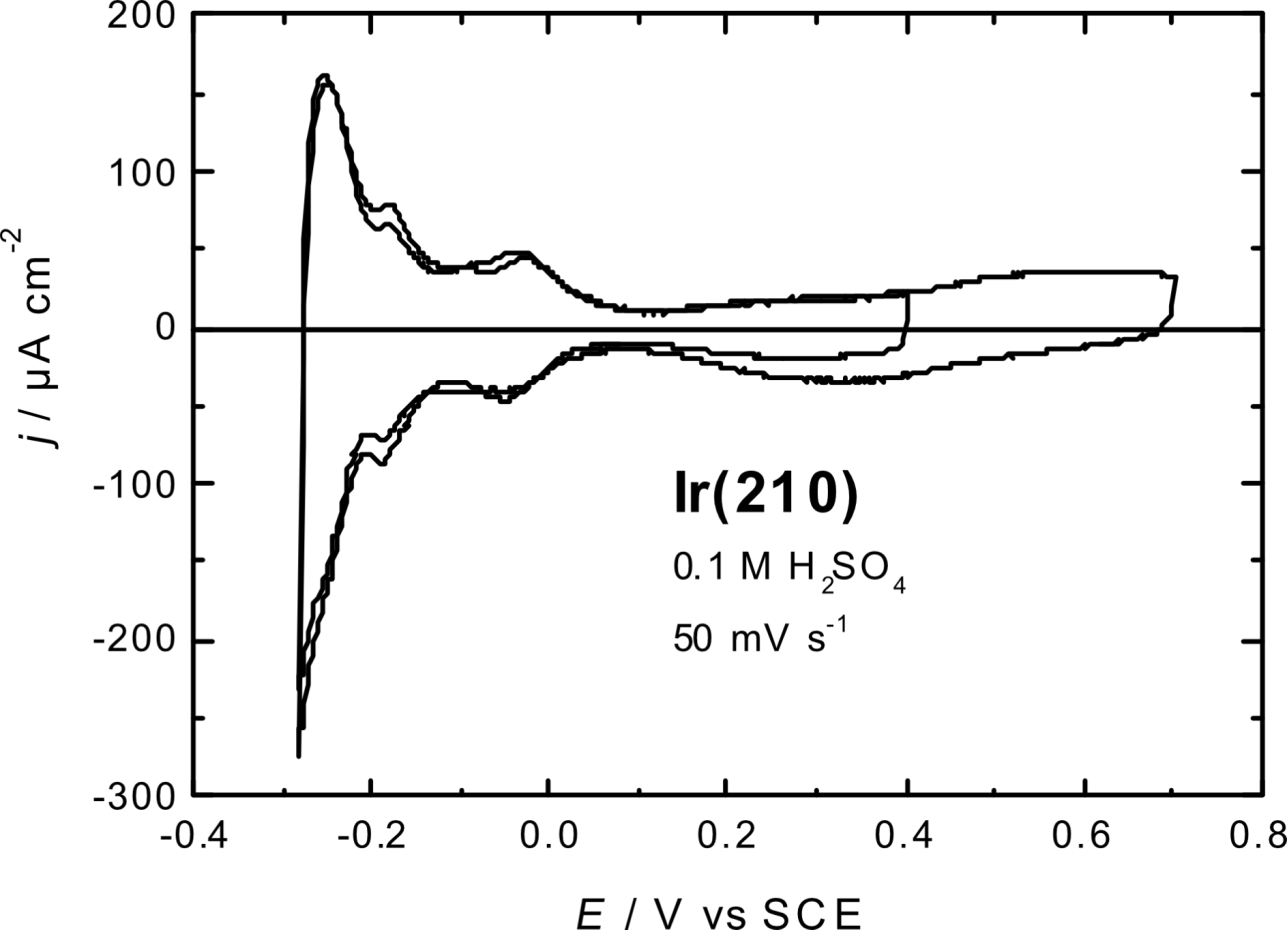
Cyclic voltammograms of Ir(210), which was annealed and cooled in a N_2_ + CO mixture, after anodic stripping of the CO adlayer in 0.1 M H_2_SO_4_. Scan rate: 50 mV·s^−1^.

Figure 1 shows typical cyclic voltammograms of the freshly-prepared Ir(210) single crystal electrode in 0.1 M H_2_SO_4_ after anodic stripping of the CO adlayer. There are three current peaks in the hydrogen adsorption region located at −0.04, −0.18 and −0.25 V. As in the case of low-index planes of Ir, these peaks are assigned to hydrogen adsorption/desorption combined with (bi)sulphate desorption/adsorption, respectively [5,17–18]. In contrast to low-index Ir surfaces [5], there are no very sharp voltammetric peaks for the relatively open (210) surface orientation.

The total charge density in the hydrogen adsorption region is around 300 µC·cm^−2^, as reported earlier [20]. Surface oxidation, including the adsorption of O/OH, starts at potentials more positive than 0.1 V. A rather broad peak for reduction of the oxidized surface is centred at 0.3 V. Stable voltammograms with reproducible hydrogen adsorption peaks were obtained by keeping the positive potential limit below 0.4 V. Annealing and cooling in the presence of CO leads to very similar current–potential curves as for H_2_-cooled Ir(210) [20], although the CO adlayer has to be removed in a single scan into the positive potential region.

### In situ STM of Ir(210) surfaces

The effect of the cooling atmosphere after annealing of noble metal single crystal electrodes has been investigated earlier for Pt(111) [24], Pt(100) and Pt(110) surfaces [6]. It was reported that the use of CO as a cooling gas for Pt(110) leads to the formation of an unreconstructed (1×1) surface [6,25], while cooling in N_2_ preserves the reconstructed Pt(110) surface [6]. The influence of the reducing cooling gases (H_2_ or CO) on the surface structure of Ir(210) in 0.1 M H_2_SO_4_ was studied by in situ STM measurements.

**Figure 2:**
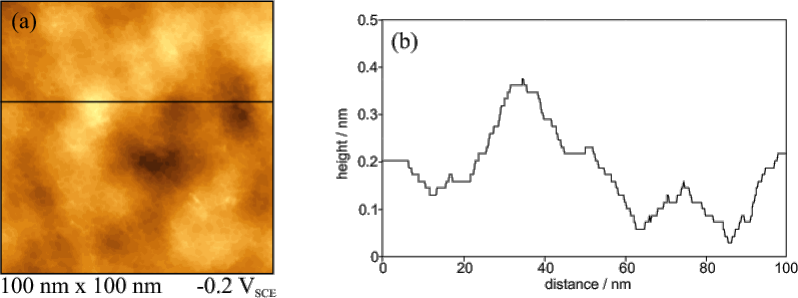
(a) in situ STM image of CO-cooled Ir(210) in 0.1 M H_2_SO_4_ at −0.2 V. (b) Height profile along the line shown in (a).

**Figure 3:**
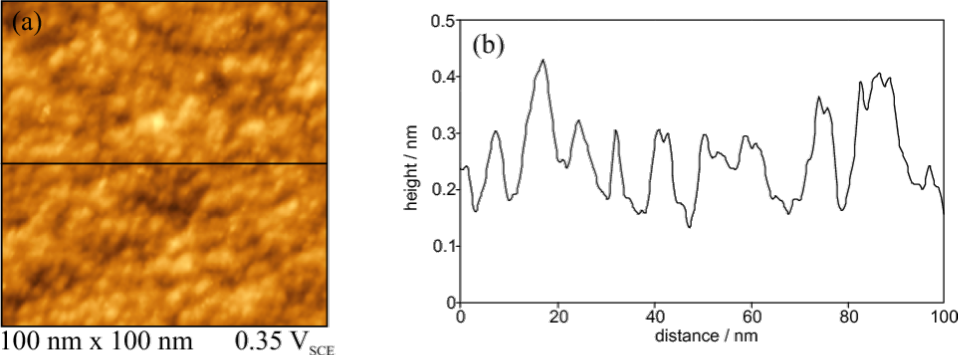
in situ STM image of H_2_-cooled Ir(210) in 0.1 M H_2_SO_4_ at 0.35 V. (b) Height profile along the line shown in (a).

Figure 2a and Figure 3a display topographic images of Ir(210) in 0.1 M H_2_SO_4_ after preparation by inductive heating and cooling down in the presence of CO and H_2_, respectively. Bright spots in these images represent higher areas, while dark ones represent lower surface regions of Ir(210). In contrast to low-index Ir surfaces such as Ir(111) [26], the STM images in Figure 2a and Figure 3a do not show wide terraces separated by monoatomic high steps. Rather small flat surface regions appear for CO-cooled Ir(210), as seen in the height profile shown in Figure 2b.

The height profile shown in Figure 3b indicates that the density of surface defects is higher for the H_2_-cooled Ir(210) surface than that for the CO-cooled surface (Figure 2b). This structural difference is not obvious from the current–potential curves in Figure 1, which are basically identical with those for Ir(210) prepared by H_2_–cooling [20]. However, it will be shown below that the cooling gas (CO or H_2_) has a strong impact on the electrocatalytic activity of Ir(210) towards CO adlayer oxidation (see below in Figure 8). The high density of atomic steps and kinks of H_2_-cooled Ir(210) is very similar to that of other high index planes, as in the case of Pt(210) [9]. Since LEED patterns obtained for a clean Ir(210) surface under UHV show a (1×1) structure [21], we assume that unreconstructed Ir(210) surfaces are also obtained after annealing and cooling in CO or H_2_.

### Potential cycling effects on Ir(210) surface

Previous theoretical studies predicted an electrochemical facet formation, i.e., potential-induced, on Ir(210) surface upon adsorption of oxygen [19]. First experiments revealed, however, that a simple polarization of Ir(210) in 0.1 M H_2_SO_4_ at potentials more positive than 0.1 V, i.e., in the region of oxygen adsorption/surface oxidation, did not lead to the expected structural changes.

**Figure 4:**
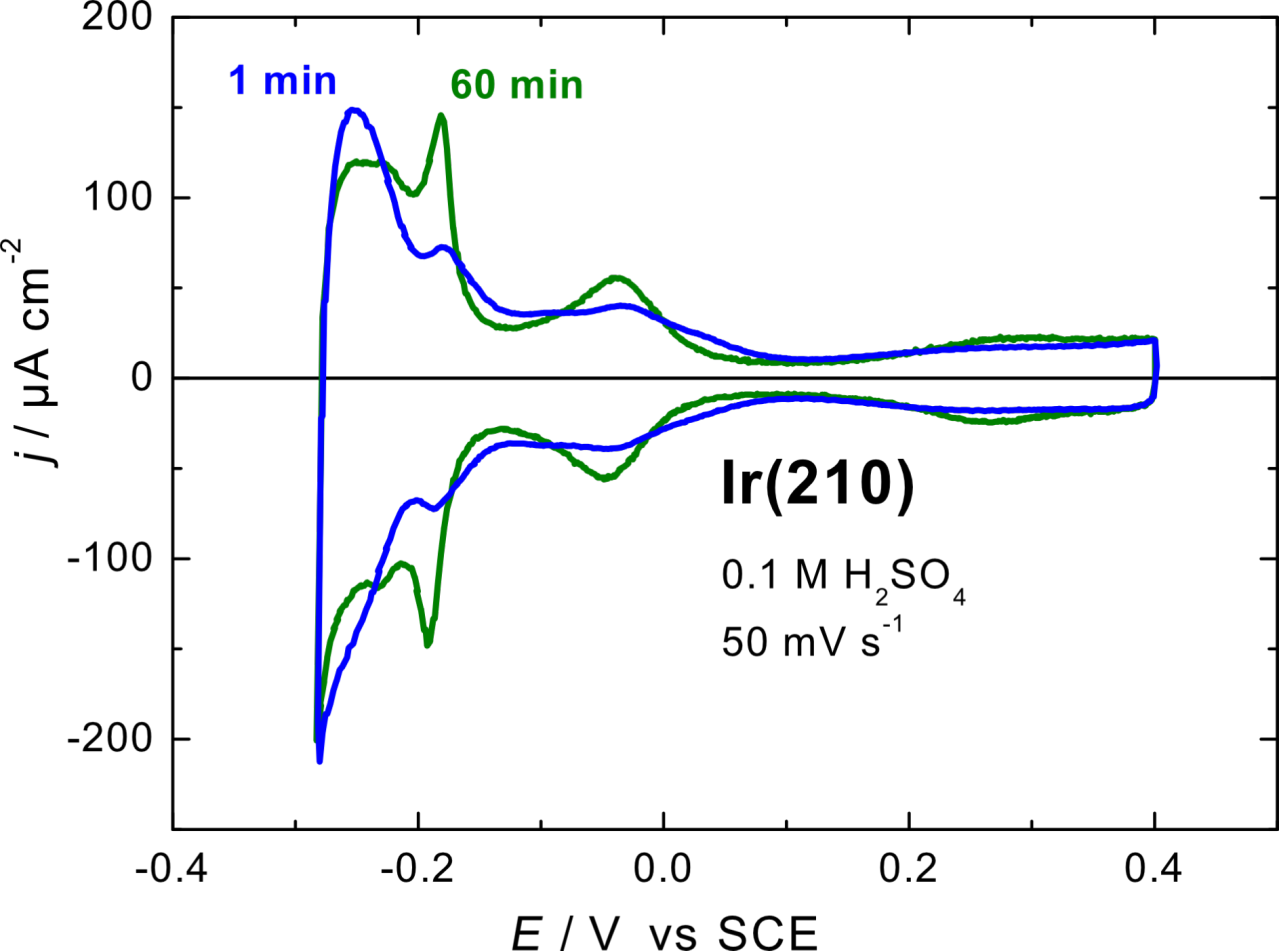
Current–potential curves for Ir(210) in 0.1 M H_2_SO_4_ after cycling at 1 V·s^−1^ between −0.28 and 0.7 V. Scan rate: 50 mV·s^−1^.

The formation of facets by potential cycling has been extensively studied with platinum [27–32], rhodium [33] and gold surfaces [34–36]. Accordingly, the Ir(210) single crystal electrode was subjected to potential cycling at scan rates between 0.05 and 2 V·s^−1^ in 0.1 M H_2_SO_4_ in the potential region between −0.28 to 0.7 V for different periods of time up to 4 h. In the following, we present results obtained with a scan rate of 1 V·s^−1^, which show the most obvious effects. As representative examples, Figure 4 shows the effect of potential cycling for 1 min and 60 min on the voltammograms of Ir(210) in 0.1 M H_2_SO_4_.

Cycling the Ir(210) electrode for 1 min caused only slight changes in the voltammogram, as can be seen by comparing Figure 4 with Figure 1. However, the voltammogram of the Ir(210) electrode subjected to 60 min of potential cycling showed an increase of the peak current intensities at −0.18 and −0.04 V concurrently with a decrease of the peak current at −0.25 V. The noticeable increase in the former two peaks (at −0.18 and −0.04 V) indicates the possible formation of (311) facets [20], because the peak at −0.18 V is characteristic of Ir(311) [5].

**Figure 5:**
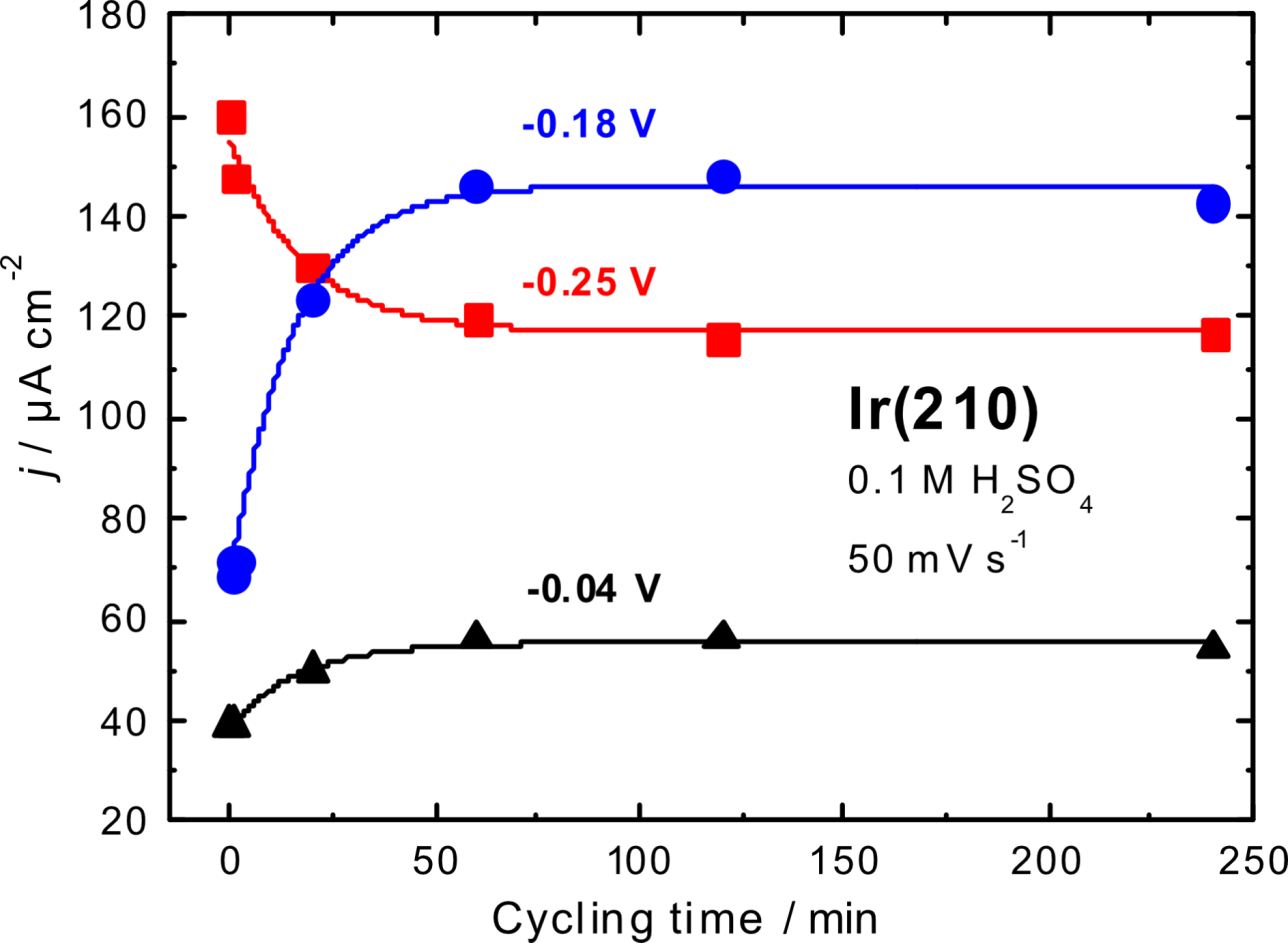
Current densities for hydrogen adsorption peaks of Ir(210) in 0.1 M H_2_SO_4_ as a function of the potential cyclic time. The curves show non-linear fits according to first order laws.

Figure 5 displays quantitative changes in hydrogen adsorption peak heights as a function of the cycling time. This graph demonstrates formal kinetics of facet formation or surface restructuring of Ir(210) in 0.1 M H_2_SO_4_. The increase of the peak current at −0.18 V is a good indication for (311) facet formation, as mentioned above. The charge densities in the hydrogen adsorption region of Ir(210) electrode after 1 min of potential cycling are practically the same as for the freshly-prepared Ir(210) electrode (300 µC·cm^−2^), although both surfaces before and after cycling have different structures as depicted by the in situ STM images (see below).

**Figure 6:**
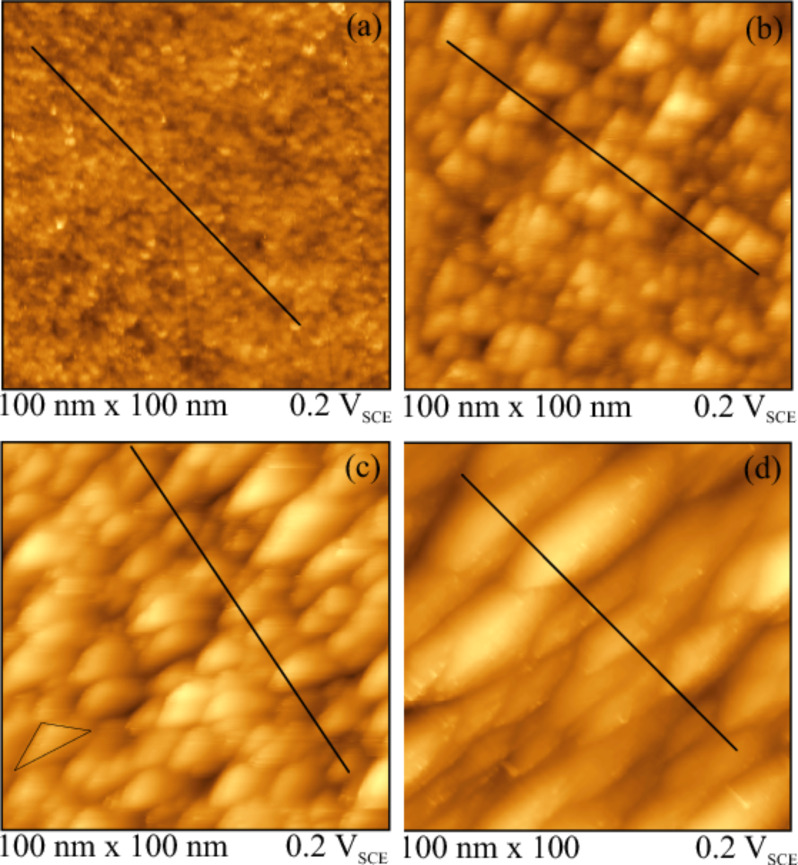
In situ STM images (100 × 100 nm^2^) of CO-cooled Ir(210) surface in 0.1 M H_2_SO_4_ after cycling between −0.28 and 0.7 V at 1 V·s^−1^ for (a) 1, (b) 20, (c) 60 and (d) 240 min.

A significantly higher charge density (345 µC·cm^−2^) was determined for the hydrogen adsorption region of the Ir(210) crystal after 60 min of potential cycling. This is a clear indication for changes in the morphology and roughness of the Ir(210) surface. Only very slight changes were seen in the voltammograms of Ir(210) for cycling times longer than 60 min (Figure 5). Nevertheless, there are still detectable changes in the surface structure of Ir(210) after longer cycling times (see below in Figure 6), which escape from the voltammetric analysis.

As mentioned above, potentiostatic polarization of a freshly-prepared Ir(210) electrode for example at 0.7 V did not lead to changes in the hydrogen adsorption peaks. While a sufficiently high coverage of oxygen species should be obtained under these conditions, potential cycling provokes the desired movement of surface atoms [36]. In addition, though faceting is thermodynamically driven, it is hindered (and limited) by the kinetic barriers involved in the atom rearrangement at the surface [37]. Thus, not only a critical adsorbate (here oxygen) coverage is required but also appropriate activation, allowing the system to overcome the kinetic barriers in the process of facet formation [23]. The voltammetric peak at −0.18 V for Ir(210) after potential cycles, which indicates (311) facets, is not as sharp as that of thermally-induced faceted Ir(210) [20]. While thermal activation is effective, electrochemical activation by potential cycling at room temperature seems to work, however less pronounced or less well-defined. Electrochemical treatment including potential cycling of Ir(210) in 0.1 M HCl did not lead to comparable changes, probably because adsorbed chloride hinders oxygen adsorption.

### In situ STM of Ir(210) after repetitive fast potential cycles

The change in surface topography of Ir(210) by repetitive oxidation–reduction potential cycles has been investigated by using in situ STM. Figure 6 shows the corresponding images of Ir(210) in 0.1 M H_2_SO_4_ after cycling for 1 min, 20 min, 60 min and 240 min. The series of STM images indicates that the surface morphology is gradually changing with cycling time. Already after 1 min of potential cycling (Figure 6a), the surface becomes rougher compared to the untreated surface (Figure 2). Cycling for 20 min leads to the formation of small triangular structures (Figure 6b), which resemble the well-defined surface structure of thermally faceted Ir(210) [20].

**Figure 7:**
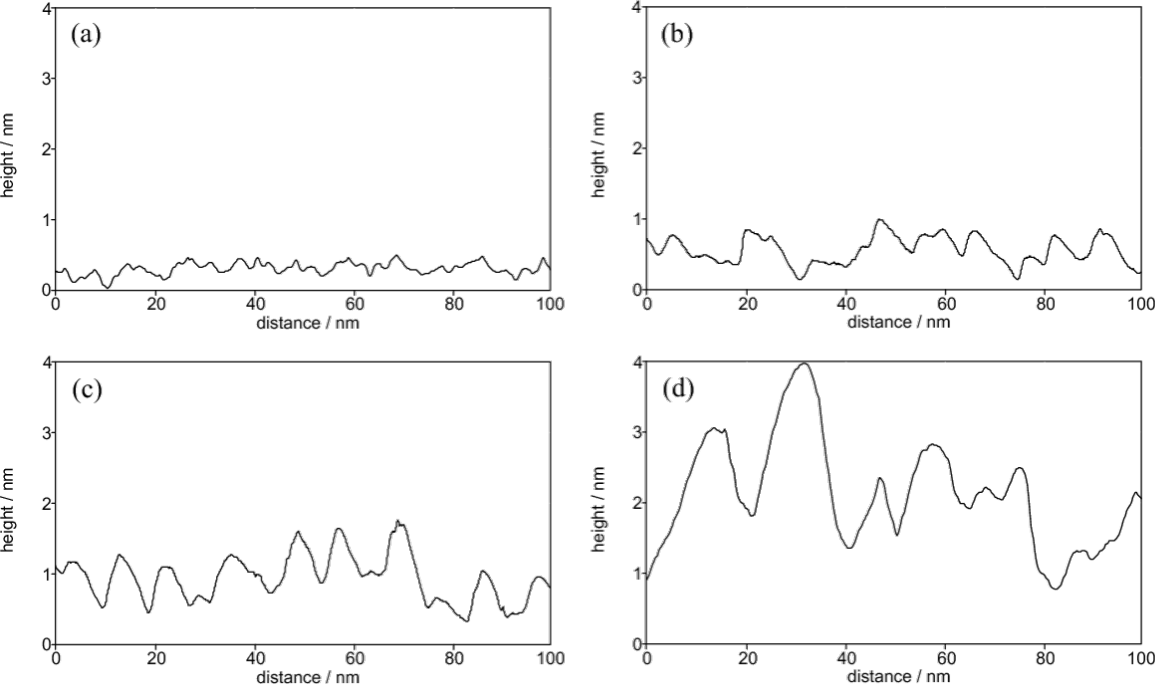
Height profiles of the CO-cooled Ir(210) surface in 0.1 M H_2_SO_4_ after cycling between −0.28 and 0.7 V at 1 V·s^−1^ for (a) 1, (b) 20, (c) 60 and (d) 240 min.

Ermanoski et al. showed that for the thermally-faceted Ir(210) surface the angles between the pyramidal faces and the (210) substrate obtained by LEED are in good agreement with the theoretical tilt angles of 19.3° and 18.4° for (311) and (110) facets, respectively [22]. While these tilt angles of the facets were verified experimentally both under UHV and electrochemical conditions [20–21], the presence of a superstructure on Ir(110) facets consisting of a stepped double-missing-row reconstruction also leads to a smaller tilt angle of only around 7° [21,37]. Figure 7 presents the cross section profiles for the potential-induced faceted Ir(210) surface along the solid lines marked in Figure 6. The lines scans indicate that the Ir(210) surface is completely facetted electrochemically. Since the tilt angles range from 6 to 28° and show clear variations in a single STM image, the electrochemically facetted Ir(210) surfaces are not as well-defined as the faceted surfaces obtained after thermal activation.

Increasing the cycling time to 60 min resulted in the formation of larger triangular structures (see black triangle in Figure 6c), which cover the whole Ir(210) surface (Figure 6c). These triangular structures are very similar to the thermally-induced faceted Ir(210) surface [19–20] and to samples prepared under UHV conditions [22]. For the case of 240 min potential cycling, anisotropic groove structures are formed that seem to be even more stable than the triangular structure under the chosen experimental conditions (Figure 6d). However, the two CVs of Ir(210) in sulfuric acid after 60 and 240 min of potential cycling are practically indistinguishable. Still, we compared our cyclic voltammograms (e.g., those in Figure 4) obtained by potential cycling to those of extended vicinal Ir single crystal surfaces [5]. There are striking similarities with Ir(320), Ir(310) and Ir(410), for example.

### CO adlayer oxidation on Ir(210)

Studies of CO oxidation on single crystal electrodes are of practical as well as fundamental interest. From the electrocatalytic point of view, CO is the most prominent intermediate species responsible for the poisoning of metallic catalysts [38]. Understanding the mechanism of the CO oxidation on single crystal electrodes may lead to a deeper insight into the relation between surface structure and electrocatalytic activity. Therefore, we chose carbon monoxide as a structure-sensitive probe of the electrocatalytic activity of restructured Ir(210), subjected to potential cycles.

**Figure 8:**
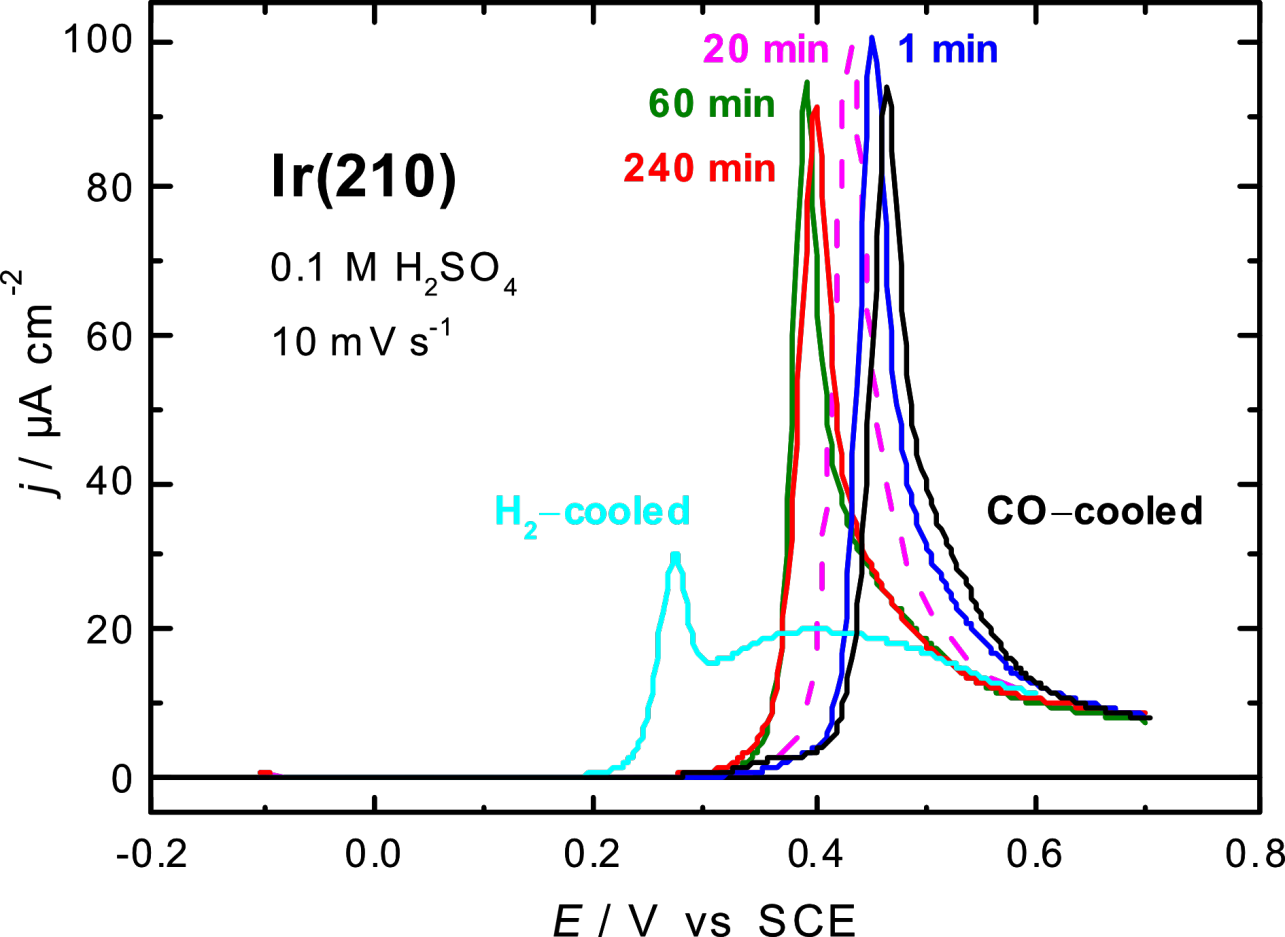
Current–potential curves for CO adlayer oxidation on Ir(210) in 0.1 M H_2_SO_4_ before and after changing the surface morphology by potential cycling. Scan rate: 10 mV·s^−1^.

Figure 8 shows linear sweep voltammograms for CO adlayer oxidation at Ir(210) in 0.1 M H_2_SO_4_ before and after the potential cycling treatment. The peak potential for CO adlayer oxidation on the CO-cooled Ir(210) electrode lies at 0.46 V (Figure 8), whereas there are two distinct oxidation peaks at 0.25 V and 0.4 V for the H_2_-cooled Ir(210) electrode (Figure 8). It was supposed that diffusion of reaction partners may be involved in the oxidation reaction mechanism [19]. So far, we were not able to identify the type of surface defects, which act as active centers for CO oxidation on H_2_-cooled Ir(210). However, these sites are absent at the CO-cooled Ir(210) surface, which explains the higher overpotential. After applying oxidation–reduction cycles, the peak potential for the CO adlayer oxidation is shifted to lower values compared to the CO-cooled Ir(210) (Figure 8). The peak potentials for CO adlayer oxidation are 0.45 V, 0.43 V, 0.39 and 0.4 V after potential cycling for 1, 20, 60 and 240 min, respectively. Thus, the restructured, electrochemically facetted Ir(210) surfaces are clearly more active than the planar Ir(210) electrode obtained by inductive heating and subsequent cooling in CO atmosphere. Several explanations might be suggested to account for the observed behaviour. Among the important parameters is the change in binding energy of adsorbed CO from 2.46 eV on Ir(210) to 2.19 eV on Ir(311) [39]. This should be directly reflected by the relatively facile oxidation of CO at the facetted surface enriched with (311) faces compared to the untreated planar Ir(210) electrode. Another reason attributed to the enhancement is the structural change produced by the oxidation–reduction cycles, resulting in an enrichment of oxygen-containing species on the surface at lower overpotentials, which is essentially required for CO oxidation. The structural changes are very clear from the in situ STM image of Ir(210) surface before and after potential cycling, see Figure 2 and Figure 6, respectively.

It should be mentioned that the Ir(210) electrode, annealed and cooled down in hydrogen atmosphere, is far more active than the restructured Ir(210) surfaces presented here and is also more active than the thermally-induced faceted Ir(210) surfaces [19]. However, so far we could not identify the reactive sites, which enhance CO adlayer oxidation significantly. It is also seen in Figure 3, that the density of defects on the H_2_-cooled surface is higher than on the CO-cooled one. This is also in agreement with the results obtained under an UHV system in which a planar Ir(210) surface was found to be more active than faceted Ir(210) for CO oxidation to form CO_2_ [40]. CO adlayer oxidation turns out to be much more structure-sensitive than the electrochemical processes taking place in dilute sulfuric acid, for example hydrogen adsorption. We would like to mention that potential cycling of the H_2_-cooled Ir(210) lead to similar electrochemical surface faceting as for the CO-cooled Ir(210) surface.

## Conclusion

In the present study, we have explored electrochemical facet formation on Ir(210). Potential cycling of Ir(210) single crystal electrode in 0.1 M H_2_SO_4_induces surface restructuring. Different structure types are forming as a function of cycling time. Triangular structures are obtained after 20 min and/or 60 min of potential cycling between −0.28 and 0.7 V, while an anisotropic groove structure is formed after 240 min. The restructured Ir(210) surfaces are more active towards the CO adlayer oxidation than planar Ir(210), which has been prepared by inductive heating and cooling in CO atmosphere. Annealing of Ir(210) and cooling in the presence of hydrogen leads to the most active surface for CO adlayer oxidation in this study. The enhanced electrocatalytic activity is probably related to a lower CO binding energy, a higher surface roughness and a larger amount of defect sites on the faceted Ir(210) surface. The results verify the theoretical prediction that faceting of Ir(210) is possible under electrochemical conditions.
